# Editorial: The role of autophagy in infectious diseases, volume II

**DOI:** 10.3389/fcimb.2025.1764139

**Published:** 2026-01-05

**Authors:** Hua Niu, Marc Herb, Joaquin Miguel Pellegrini

**Affiliations:** 1 Laboratory of Hepatobiliary and Pancreatic Surgery, The First Affiliated Hospital of Guilin Medical University, Guilin, China; 2 Guangxi Key Laboratory of Molecular Medicine in Liver Injury and Repair, Guilin Medical University, Guilin, China; 3 Guangxi Health Commission Key Laboratory of Basic Research in Sphingolipid Metabolism Related Diseases, The First Affiliated Hospital of Guilin Medical University, Guilin, China; 4 Faculty of Medicine and University Hospital of Cologne, Institute for Medical Microbiology, Immunology and Hygiene, Cologne, Germany; 5 Aix Marseille Université, CNRS, INSERM, Centre d’Immunologie de Marseille-Luminy (CIML), Marseille, France

**Keywords:** autophagic regulation, autophagy, host defense, infectious diseases, pathogens

Autophagy is an intracellular catabolic process that sequesters and degrades cytosolic components to maintain cellular homeostasis under stress conditions (Dikic and Elazar, 2018). Based on the mechanisms by which cellular materials are delivered to lysosomes, autophagy is primarily classified into three types: macroautophagy, microautophagy, and chaperone-mediated autophagy (CMA) (Boya et al., 2013). Macroautophagy, the main autophagic pathway, involves the formation of double-membraned autophagosomes. This process is initiated by a set of evolutionarily conserved autophagy-related (ATG) proteins, including the ULK1 complex, Beclin-1, and ATG8/LC3 family proteins in the cytosol. Autophagosomes engulf damaged organelles, protein aggregates, or pathogens and subsequently fuse with lysosomes for degradation and recycling of their contents. When specifically targeting pathogens, macroautophagy is termed xenophagy (Sharma et al., 2018). Upon exposure to the cytosol, pathogens are recognized by adapter proteins such as p62/SQSTM1 and optineurin (Optn), which bind to ubiquitinated microbial proteins and recruit autophagy-related proteins like LC3 to initiate autophagosome formation around the invader (Zheng et al., 2009; Wild et al., 2011). The encapsulation and subsequent degradation of pathogens within autolysosomes restrict intracellular microbial replication, establishing xenophagy as a crucial host defense mechanism.

Besides clearing intracellular pathogens, autophagy intersects with the immune system at various stages, shaping both innate and adaptive responses. Alterations in autophagic pathways have been linked to inflammatory conditions, including autoimmune disorders and neurodegenerative diseases (Deretic, 2021). By delivering pathogen-derived antigens to major histocompatibility complex (MHC) molecules for antigen presentation, autophagy participates in the adaptive immune response (Schmid et al., 2007), while regulation of pro-inflammatory cytokine production contributes to modulate the inflammatory response (Deretic, 2021). Meanwhile, macroautophagy itself is a tightly regulated process, primarily modulated by the kinase mammalian target of rapamycin (mTOR), which integrates signals from various pathways. One well-studied regulatory pathway is the phosphatidylinositol 3-kinase (PI3K)/AKT cascade, which can be activated by ligand-receptor interactions at the cell membrane to modulate autophagy (Glaviano et al., 2023).

As aforementioned, autophagy plays a vital role in defending against intracellular pathogens. Nonetheless, many pathogens have evolved sophisticated strategies to evade or subvert autophagy, such as inhibiting autophagosome formation or preventing their maturation into autolysosomes. This Research Topic collects a series of research articles and reviews on recent advances in understanding the interactions between autophagy and bacterial or viral pathogens.

*Mycobacterium marinum* (Mm), a nontuberculous mycobacterium, causes a systemic TB-like disease in zebrafish. When Mm invades the cytosol, its surface proteins become ubiquitinated and are recognized by sequestosome 1-like receptors (SLRs). Previous studies indicated that two SLRs, Optn and p62, are important for innate host defense in zebrafish during mycobacterial infection. DNA damage regulated autophagy modulator 1 (Dram1) is a stress-inducible protein that regulates autophagy and has been shown a protective role against Mm in zebrafish. The research article by Xie et al. further investigated the complementarity and functional differences between Optn and p62, and interdependency between the two SLRs and Dram1. The authors demonstrated that overexpression of Optn, p62, or Dram1 each can rescue phenotypes of other mutants, suggesting that Optn, p62, and Dram1 function independently in the anti-mycobacterial xenophagy pathway.

Autophagic activity is regulated by multiple signaling pathways, such as the PI3K/AKT/mTOR axis. A research article by Zhang et al. revealed that during infectious bursal disease virus (IBDV) infection, autophagy is regulated via the PI3K/AKT/FoxO1 pathway. IBDV causes massive destruction of B lymphocytes in the bursa of Fabricius in chickens, leading to infectious bursal disease. The authors found that IBDV infection upregulates the expression of programmed death-1 (PD-1), an immunoinhibitory receptor, which in turn induces autophagy through the PI3K/AKT/FoxO1 pathway to promote viral replication. This work unveils a novel link between the immune system, autophagy, and viral infection.

Although autophagy serves as an important host defense mechanism against intracellular pathogens, many bacteria have evolved strategies to evade or manipulate it to promote their own replication and persistence. Two review articles by Zhang et al. and Pavlik et al. summarized recent advances in understanding how bacterial pathogens interact with autophagy. As discussed by Zhang et al., autophagy may play a dual role during *Chlamydia trachomatis* infection: while the pathogen can induce autophagy to acquire nutrients and maintain host cell homeostasis, the fusion of autophagosomes with lysosomes inhibits its proliferation. To counteract host autophagy, many bacterial pathogens secrete effector proteins that block autophagosome formation or inhibit autophagosome-lysosome fusion. Pavlik et al. first provided a comprehensive overview of how effector proteins from more than a dozen pathogens, including *Salmonella*, *Shigella*, and *Legionella*, modulate the autophagic pathway. They then detailed the interaction between *Francisella tularensis* and host cells. The highly virulent strain *F. tularensis* subsp. *tularensis* SchuS4 evades ATG5-dependent autophagy while inducing ATG5-independent autophagy to increase intracellular pools of host amino acids, which are utilized as a carbon and energy source. Both reviews indicate the dual role of autophagy—acting both as a defense mechanism and as a resource exploited by intracellular pathogens like *C. trachomatis* and *F. tularensis*.

Collectively, this Research Topic provides updated knowledge on the interplay between host autophagy and pathogens. Further studies are needed to understand the precise role of autophagy in some bacterial infections, and the crosstalk between the immune system and autophagy in pathogenic infections.
